# Exploring the response of a key Mediterranean gorgonian to heat stress across biological and spatial scales

**DOI:** 10.1038/s41598-022-25565-9

**Published:** 2022-12-06

**Authors:** D. Gómez-Gras, N. Bensoussan, J. B. Ledoux, P. López-Sendino, C. Cerrano, E. Ferretti, S. Kipson, T. Bakran-Petricioli, E. A. Serrao, D. Paulo, M. A. G. Coelho, G. A. Pearson, J. Boavida, I. Montero-Serra, M. Pagès-Escolà, A. Medrano, A. López-Sanz, M. Milanese, C. Linares, J. Garrabou

**Affiliations:** 1 Departament de Biologia Marina, Institut de Ciències del Mar (CSIC), Barcelona, Spain; 2 Departament de Biologia Evolutiva, Ecologia i Ciències Ambientals, Universitat de Barcelona (UB), Barcelona, Spain; 3 Institut de Recerca de la Biodiversitat (IRBio), Universitat de Barcelona (UB), Barcelona, Spain; 4 CNRS, IRD, Mediterranean Institute of Oceanography (MIO) UTM 110, University of Aix-Marseille, University of Toulon, Marseilles, France; 5 CIIMAR/CIMAR, Centro Interdisciplinar de Investigação Marinha e Ambiental, Universidade do Porto, Porto, Portugal; 6 Dipartimento di Scienze della Vita e dell’Ambiente (DiSVA), Università Politecnica delle Marche, Ancona, Italy; 7 Consorzio Nazionale Interuniversitario per le Scienze del Mare (CoNISMa), Rome, Italy; 8 Stazione Zoologica Anton Dohrn, Naples, Italy; 9 Reef Check Italia Onlus, Ancona, Italy; 10 Department of Biology, Faculty of Science, University of Zagreb, Zagreb, Croatia; 11 SEAFAN-Marine Research and Consultancy, Zagreb, Croatia; 12 CCMAR, University of Algarve, Faro, Portugal; 13 Studio Associato GAIA s.n.c., Genoa, Italy

**Keywords:** Climate-change ecology, Conservation biology, Marine biology

## Abstract

Understanding the factors and processes that shape intra-specific sensitivity to heat stress is fundamental to better predicting the vulnerability of benthic species to climate change. Here, we investigate the response of a habitat-forming Mediterranean octocoral, the red gorgonian *Paramuricea clavata* (Risso, 1826) to thermal stress at multiple biological and geographical scales. Samples from eleven *P. clavata* populations inhabiting four localities separated by hundreds to more than 1500 km of coast and with contrasting thermal histories were exposed to a critical temperature threshold (25 °C) in a common garden experiment in aquaria. Ten of the 11 populations lacked thermotolerance to the experimental conditions provided (25 days at 25 °C), with 100% or almost 100% colony mortality by the end of the experiment. Furthermore, we found no significant association between local average thermal regimes nor recent thermal history (i.e., local water temperatures in the 3 months prior to the experiment) and population thermotolerance. Overall, our results suggest that local adaptation and/or acclimation to warmer conditions have a limited role in the response of *P. clavata* to thermal stress. The study also confirms the sensitivity of this species to warm temperatures across its distributional range and questions its adaptive capacity under ocean warming conditions. However, important inter-individual variation in thermotolerance was found within populations, particularly those exposed to the most severe prior marine heatwaves. These observations suggest that *P. clavata* could harbor adaptive potential to future warming acting on standing genetic variation (i.e., divergent selection) and/or environmentally-induced phenotypic variation (i.e., intra- and/or intergenerational plasticity).

## Introduction

Ocean warming is imposing increasing stress on marine ecosystems by exposing marine species to extreme temperatures that may exceed their thermal limits^1^. However, populations and individuals of the same species exhibit contrasting responses to warming across different spatio-temporal scales^2–4^. This intra-specific variability hinders our understanding of species vulnerability to climate change, and therefore, of any potential derived effect that may cascade up to the community or ecosystem level.

The capacity of sessile marine species to persist in the face of climate change is highly influenced by their thermal tolerances, which tend to reflect the environment in which they are found^5^. Accordingly, much research has focused on deciphering how past and present local environmental conditions modulate responses to ocean warming in relation to biological processes. Thermal acclimation through adaptive phenotypic plasticity (i.e., the expression of distinct phenotypes from an individual genotype, thus increasing relative fitness in response to temperature variations), and local thermal adaptation via divergent selection (i.e., selection on locally adapted genomes exhibiting higher relative fitness in their local thermal environment than foreign individuals) have received particular attention^6–9^. In coral species, populations thriving in warmer environments have often been observed to exhibit higher thermal tolerances than those living under more moderate temperature conditions, and is commonly attributed to local thermal adaptation of the coral host and/or their photo-symbionts^8,10–16^. Similarly, recent thermal history (i.e., exposure to heat stress or marine heatwaves [MHWs]) in the weeks or months prior to subsequent exposure to heat stress has also been shown to potentially modify the thermal responses of coral populations, leading to acclimation, hardening weakening effects and/or the differential survival of resistant genotypes^13,17–21^. However, while the local thermal environment and the thermal and stress histories of populations seem to play important roles in the determination of thermotolerance in some coral species, the relationship between these factors remains controversial for others (e.g.,^22–26^).

In the Mediterranean Sea, MHWs are recurrently triggering severe mass mortality events (MMEs) affecting a wide array of benthic macro-invertebrates across multiple phyla^2,27,28^. However, the effects are not homogenous, and typically result in contrasting patterns of mortality between different organisms, species and populations. Among them, the habitat-forming red gorgonian *Paramuricea clavata* has been one of the most affected species, providing an excellent biological model to explore the factors and processes modulating intra-specific responses to heat stress. In recent years, some studies have provided evidence in support of various eco-physiological factors that could influence the responses to heat stress of different *P. clavata* populations. For instance, feeding constraints prior to a MHW (typically during summer-autumn) may exacerbate MMEs in affected populations by inducing a metabolic imbalance between the higher energetic cost of respiration at high temperatures and the energetic constraints of the locally weakened colonies^29–31^. Alternatively, the local abundance of thermo-dependent and/or opportunistic pathogens may also contribute to the MHW-driven mortality of debilitated *P. clavata* populations^32,33^. A recent study indeed reported increases of both opportunistic and pathogenic bacteria in the microbiome of Mediterranean gorgonians during thermal anomalies^34^ (but see^35^). Demographic factors (e.g., sexual maturity, sexual condition) could also partially explain why individuals and populations from the same and different locations show contrasting responses to thermal stress^36^. Yet, despite the fundamental insights provided by these and other previous studies*,* little is yet known about the potential role that local thermal environments and thermal and stress histories could play in shaping intra-specific variation in *P. clavata* thermotolerance. This hinders our understanding of the overall thermal sensitivity of this species, as well as of its future capacity to adapt and/or acclimatize to warmer conditions via genetic and/or physiological mechanisms.

Nevertheless, some efforts have already been made in this direction. In a previous study of thermotolerance among different NW Mediterranean *P. clavata* populations^25^, tolerance was correlated with genetic drift which, by restricting the capacity for local adaptation, was suggested as a driver of the variation observed in response to MHWs. A central role for genetic drift in the response to thermal stress of *P. clavata* was also suggested by population genetic data^37^. Beyond this previous study, the effects of thermal stress in *P. clavata* have only been examined to date in closely related individuals and/or populations, either from the same location but at different depths, or from a few relatively nearby locations within the same sub-basin in the Mediterranean Sea (maximum distance approximately 500 km). Moreover, the potential influence of prior thermal exposure on the overall outcomes remains an open question.

In this study, we performed a common garden experiment in aquaria encompassing a spatial scale not previously addressed (> 1500 km of coast) for *P. clavata*. A total of 11 populations from 4 different localities situated across the North and West Mediterranean were submitted to thermal stress in controlled conditions. Our aims were threefold: (1) to test the null hypothesis that warmer thermal regimes do not result in better thermotolerance (marginal role for local adaptation in thermal stress response); (2) to test whether the sea water temperature conditions in the months prior to the experiment (i.e., a proxy for recent thermal history) influenced the response, which could indicate the capacity for rapid acclimation and/or weakening effects; and (3) to explore the potential effect of previous MHW-induced MMEs (i.e., proxy for longer-term thermal stress history) on thermotolerance, which could provide further insights into the role that extreme warm events may have on the response of *P. clavata* to recurrent MHWs. Taken together, this information will contribute to increase our knowledge on the adaptive potential of *P. clavata* to climate change, considered here as the capacity to tolerate or adapt to future conditions via adaptive phenotypic (intra or trans-generational acclimation processes) and/or genetic changes (adaptation via natural selection). The results obtained will contribute to the conservation of this emblematic Mediterranean species, including the potential identification of thermoresistant donor populations and/or colonies for future restoration actions.

## Materials and methods

### Model species

The red gorgonian *Paramuricea clavata* is a habitat-forming octocoral with a key role in the structure and functioning of temperate hard-bottom habitats such as the coralligenous assemblages^38–40^. Distributed in dim light conditions along the Mediterranean coasts^41^, *P. clavata* is characterized by its arborescent morphology, great longevity (up to 100 years) and notable size (up to 2 m)^42^. Thus, *P. clavata* presents a unique set of traits that increases habitat complexity and provides ideal environmental conditions favoring the settlement and development of many other species of the coralligenous^38^. However, this species also has traits that make it especially sensitive to disturbances. With slow growth, low recruitment rates and restricted dispersal^37,43–46^, *P. clavata* is increasingly threatened by anthropogenic impacts that cause mortality in adults. These include physical damage and mortality from fishing activities^47^, invasive species^48^, habitat degradation^49^ and climate change^27,28,40,50^. Accordingly, *P. clavata* was included in the IUCN red list of vulnerable Mediterranean Anthozoans in 2016^51^.

### Study locations

**Figure 1:**
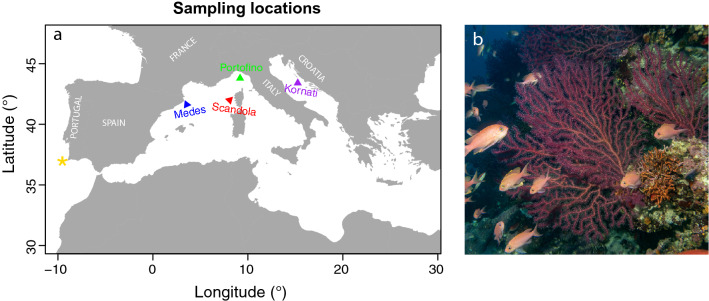
(**a**) Location of the four sampling localities across the Mediterranean. * Another population from Sagres (Portugal) was initially included in the thermal experiment, but was withdrawn from the analyses after recently confirmed to be a different species of *Paramuricea.* (**b**) Photo of the study species *Paramuricea clavata (photo by Medrecover).* The map shown in panel (**a**) was generated using R software version v3.5.0 (https, //www.R-project.org/) and combined with the photo in panel (**b**) using Adobe Illustrator CC 2018 (https://adobe.com/products/illustrator).

**Figure 2:**
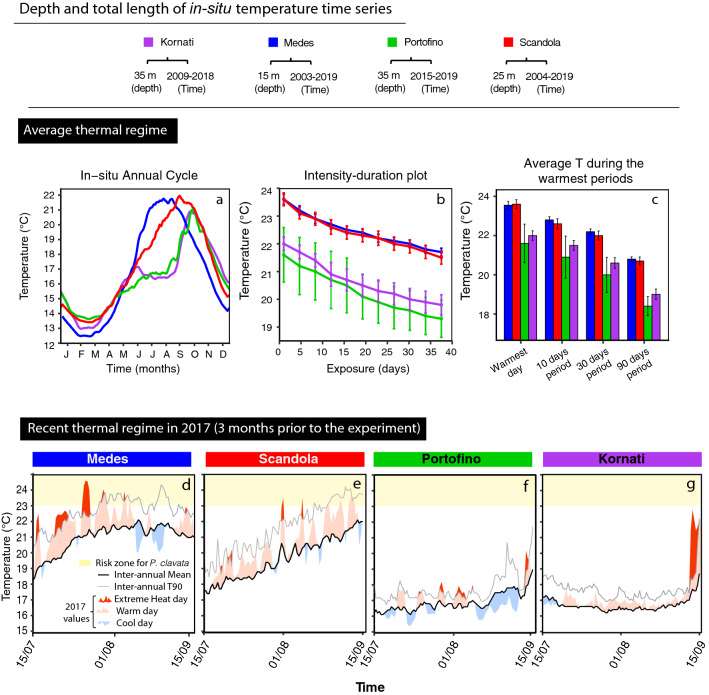
(**a**) Inter-annual average of daily mean temperatures during the annual cycle at each locality at the depths on which the populations were sampled. (**b**) Intensity-duration plot indicating the mean number of days (± SE) at warm temperatures during the 10% warmest period of the year at each locality. (**c**) Mean locality temperature (± SE) during the warmest day, 10-days, 30-days and 90-days of each year. (**d**–**g**) Daily temperature values observed *in-situ* at each locality during the three months prior to the experiment (from 15th June until 15th September, 2017) with respect to the inter-annual mean (iMean) and the inter-annual percentile 90th (iT90). Days over the iT90 threshold were considered as “extreme heat days”. Days over the iMean but below the iT90 were considered as “warm days”. Days below the iMean were considered as cool days. Finally, the risk zone for *P. clavata* (days over 23 °C) is represented in yellow.

Samples were taken from 11 *P. clavata* populations located within four different localities situated across the North and West Mediterranean basin and the Adriatic Sea (Fig. 1 and Table 1) based on the following criteria: (1) large spatial scale (hundreds to thousands of kilometres), encompassing large longitudinal (from 3 to 15° E) and latitudinal ranges (from 42 to 44° N), which is representative of the species geographic distribution, (2) contrasting average *in-situ* thermal regimes at the depths sampled (the upper limit of the bathymetric range of *P. clavata* in each locality) (see Fig. 2a–c in “Results” section), (3) local temperature variation in the three months prior to the experiment, with some localities exhibiting abnormally high temperatures, which may have pre-conditioned *P. clavata* colonies for the thermal experiment (i.e. contrasting recent thermal history; see Fig. 2d-g in “Results” section); and (4) encompassing a range of previous local MHW-induced MMEs (i.e., contrasting thermal stress history; Table S1).

**Table 1:** Detailed information on the sampled populations; country, location, ID, coordinates and sampling depths.

Country	Localities	Population ID	Longitude	Latitude	Depth (m)
Spain	Medes (Catalonia)	La vaca	3° 13′ 34.76'' E	42° 2′ 52.97'' N	18—20
		Pota del Llop	3° 13 ′31.44' 'E	42° 2′ 58.92'' N	15 -17
		Tascons	3° 13′ 36.84ʺ E	42° 2′ 31.88ʺ N	15 – 17
France	Scandola (Corsica)	Gargallu	8° 32′ 3.82ʺ E	42° 22′ 18.62ʺ N	24 – 27
		Palazzinu	8° 33′ 0ʺ E	42° 22′ 47.47ʺ N	23—26
		Palazzu	8° 32′ 46.19ʺ E	42° 22′ 47.54ʺ N	23–26
Italy	Portofino (Liguria)	Altare	9° 10′ 43.12ʺ E	44° 18′ 32.10ʺ N	35—37
		Indiano	9° 10′ 0.80ʺ E	44° 18′ 44.91ʺ N	35 -37
		Lighthouse	9° 13′ 8.45ʺ E	44° 17′ 55.14ʺ N	35–37
Croatia	Kornati (Dalmatia)	Balun	15° 15′18'' E	43° 48′ 14'' N	33—36
		Mana	15° 15′ 59'' E	43° 48′01'' N	35

In addition to the 11 Mediterranean populations, a population from Sagres, southern Portugal (NE Atlantic) was initially included in the thermal experiment (see Supplementary Appendix S1). However, a recent phylogenomic analysis has confirmed that the specimens from this population are a different species of *Paramuricea* [Coelho et al. *in prep*]*.* Therefore, the response of this population to heat stress has been withdrawn from the analyses and discussion of the present work but is provided as Supplement (see Supplementary Appendix S2).

### Thermal environments

The subsurface thermal environment of the four Mediterranean localities was monitored as part of the T-MEDNet initiative^52^. The local temperature conditions were documented based on a standard protocol for the acquisition of multi-year time series with temperature records collected every hour at 5 m intervals between the surface and 40 m depth. To analyse the thermal environment experienced by *P. clavata* populations at the sampling depths, all corresponding *in-situ* temperature data available up to 2019 were retrieved from T-MEDNet database (from 4 to 18 years, see Fig. S1). Our comparative analysis considered the timing and magnitude of the annual daily temperature cycle and the exposure to warm conditions. Three metrics of average local thermal regime (calculated over the entire time-series), and one metric of recent thermal regime (calculated over a 3-month period prior to the experiment) were calculated to contrast with the thermotolerance responses (see Table 2 for a description of the thermal metrics).

**Table 2:** Description of the thermal metrics used to compare thermal environments.

Thermal regime	Thermal metric	Thermal metric description
Average local thermal regime	Mean annual temperature	Inter-annual average of mean daily temperatures during a year
	Mean Tmax during warm periods	Inter-annual average of Maximum temperature during the warmest periods of the year (measured at 1day, 10 days, 30 days and 90 days periods)
	N of days with high temperatures	Inter-annual average of number of days at temperatures higher than 23 °C
Recent thermal regime (3 months prior to the experiment)	N of extreme heat days	N of extreme heat days (days with T over the inter-annual percentile 90th based on the local climatology) and with a daily average temperature of at least 23 °C

### Previous exposure to MHW-induced MMEs

Data on previous MHW-induced MMEs experienced by the 11 studied populations from 1983 to 2017 were retrieved from the T-MEDNet mass mortality database^53^. Following the same classification used in the database, we considered populations exhibiting less than 30%, between 30 and 60% or more than 60% of affected colonies as experiencing low, intermediate and severe impacts, respectively. The resulting information regarding years, locations, depths, and severity of impacts (i.e., % of affected colonies) can be found in Table S1.

### Thermotolerance experiment

Field sampling was carried out by collecting apical colony branches of at least 10 cm in length from 30 healthy adult colonies (> 50 cm in height) per population (330 fragments in total) (Fig. S2). The sampling was conducted simultaneously in all localities by scuba-diving for two days (18th and 19th of September 2017). The samples were randomly selected among the colonies occurring near the upper limit of the bathymetric range of *P. clavata* at each location (ranging from 15 to 37 m depth; see Table 1). After collection, live samples were maintained overnight in a small aquarium system with recirculating seawater, packed in 2 L bags of seawater (10 fragments per plastic bag) and sent in polystyrene boxes (3 bags per box) on the 19th of September to Barcelona. In the case of Medes and Scandola, samples were directly transported in coolers immediately after sampling without overnight stabilization. Overall, the minimum and maximum transportation times between the sampling and the arrival facility was several hours for the Medes populations and 36 h in the case of populations from Portofino.

The experiment was conducted in the Aquarium Experimental Zone of the Institut de Ciències del Mar in Barcelona (ICM-CSIC) starting on the 21^th^ of September 2017. Upon arrival, each of the 30 colony branches sampled per population were divided into two fragments (8–10 cm length; one for control, one for treatment), that were mechanically fixed to experimental rectangular PVC plates (5 × 30 cm) and two rubber layers following the methodology applied by^25^. The samples were acclimated for one week in an open aquarium system with 50 μm sand-filtered running seawater at a natural temperature (17–18 °C). Colony health was checked during the week-long acclimation period before the experiment by assessing their overall appearance (i.e., presence of tissue necrosis) and polyp aperture during feeding events. All colonies presented open polyps during feeding events and showed no signs of tissue necrosis over this period. Since populations from different regions also exhibited equivalent levels of polyp aperture during feeding events (Fig. S3), we concluded that transportation did not have any relevant effect on the health status of the colonies before the experiment (as observed in previous studies on the same species and with similar transportation times, e.g.,^25^).

The common garden experiment involved two aquaria sets: control and Treatment, each of which comprised three replicate tanks (70 L each) per population with 10 different individuals in each (30 individuals per population and experimental condition). In the Control set, seawater temperature was maintained at 16–18 °C during the whole experiment. In the Treatment set, the heat stress consisted of a stepwise temperature increase from 18 to 25 °C over 3 days (Fig. S4). Upon reaching 25 °C, thermal conditions were kept constant for 25 days. This temperature was chosen for our experiment because it was identified as a lethal threshold for *P. clavata* populations^25^. In addition, a buffer tank (also 70 L) was used to control seawater temperature in both Treatments and Control tanks. The buffer tank was supplied with 50 μm sand-filtered Mediterranean seawater pumped from 15 m depth directly into the experimental tanks, functioning as an open system (Fig. S4). To monitor water temperature in each tank throughout the experiment and to facilitate circulation, each tank was provided with individual heaters, temperature controllers, HOBO temperature data loggers (accuracy 0.2 °C, resolution 0.02 °C, registering temperatures every 5 min) and submersible water pumps. Finally, feeding was carried out three times per week by combining 3 ml of a liquid mixture of particles between 10 and 450 μM in size (Bentos Nutrition Marine Active Supplement, Maim, Vic, Spain) in each tank on days 2 and 6 and a tablet of frozen cyclops (Ocean nutrition, Antwerp, Belgium) on day 4^25,54^.

### Response variables

The response variable measured as a proxy for colony thermotolerance was the percentage of tissue necrosis, which was visually monitored daily throughout the duration of the experiment^25^. From this variable, we calculated four descriptors that were used to statistically compare differences in thermotolerance among colonies, populations and localities: (1) the survival probability of each colony through time (i.e., its probability of having less than 100% of injured surface), (2) the mean (± SD) daily % of extent of injury, estimated as the daily average (or SD) injured tissue per colony in each population, (3) the daily percentage of affected colonies (those with > 10% necrotic tissue), and (4) the daily percentage of dead colonies. These descriptors are good estimators of the magnitude, timing and variability of the partial and/or total mortality suffered by *P. clavata* and other coralligenous species exposed to thermal stress (e.g.,^2,24,25,27,54^).

### Statistical analysis

To test for significant relationships between local thermal regime, recent thermal history, thermal stress history and thermal tolerance, we used a series of different mixed effects COX models^55^. These models allowed us to compare time to colony death (dependent variable) as a function of thermal descriptors (independent variables) while accounting for any potential confounding factors related to the populations or localities involved. In this sense, our different computed models varied in their fixed terms (thermal descriptors) depending on our research question, but maintained the dependent (i.e., survival exhibited by each colony throughout the 25 days of exposure to thermal stress) and random (populations nested within localities) variables constant. Specifically, we first tested whether the average local thermal regime of the colonies influenced their thermal response by including the Mean Annual T of the different localities as fixed effect. In this model, we excluded all other available descriptors of average local thermal regime (i.e., Mean Tmax during warm periods or N of days at T > 23 °C) because they were highly correlated with Mean Annual T, and thus their inclusion would have obscured the interpretation of the results. Secondly, we tested whether the recent thermal history of the colonies influenced their thermal response by selecting as fixed effect the number of extreme heat days (those with T over the 90th interannual percentile) reaching at least 23 °C over the 3 months prior to the experiment. Since 23 °C is both a sublethal temperature for *P. clavata* and close to the 25 °C used in the thermal experiment^25^, this metric allowed us to test the effects of anomalous extreme temperatures before the experiment (potential pre-conditioning) on population responses under experimental conditions. Finally, we tested whether prior occurrence of MHW-induced MMEs at the studied locations (a proxy for thermal stress history) influenced the thermal response of populations by including the degree of impact (from low to severe; see Table S1) exhibited during prior events as a fixed effect.

To further characterize differences in thermotolerance among the studied populations and localities regardless of their thermal environment or history, we used the Kaplan–Meier product limit method^56^ and the log-rank test^57^. We ran a first analysis to explore differences among localities. Then, we repeated the analyses considering each locality separately to explore differences among populations with the same regional origin. We then tested for differences among populations by considering all populations of the study pooled together. This analysis allowed a post-hoc pairwise Log-rank comparison to investigate differences between pairs of populations regardless of their origin. Finally, to investigate the variability of response among individuals across populations, we estimated population mean daily SD of extent of injury across the 25 days of the experiment, and grouped populations into three arbitrary categories; low variability (mean daily SD < 10%), intermediate variability (10% > mean daily SD < 15%) and high variability (mean daily SD > 15%). All statistical analyses were computed using the R functions from the ‘survival' and ‘coxme', R packages (R version v3.5.0; R Core Developer Team, 2018).

## Results

### Local thermal regimes at the study localities

The analysis of the inter-annual average *in-situ* temperature revealed contrasting patterns in both the timing and magnitude of the seasonal cycle for the four different localities (Fig. 2a). On average, the warmest conditions occurred at the end of summer in Medes and Scandola (i.e., August–September), and October at Kornati and Portofino. All localities experienced their minimum temperatures in February. Scandola was the locality exhibiting the highest mean temperature during the year (17.11 °C ± 2.9), whereas Portofino was the one with the lowest mean temperature (16.33 °C ± 2.1 SD). However, the warm tail of the temperature distribution (10% warmest percentile; 37 days), revealed the highest level of exposure to warm temperature (percentiles expressed in days) at Medes and Scandola (Fig. 2b), where up to 30 days per year were above 22 °C on average, while these temperatures were only rarely reached at Kornati or Portofino. Similarly, the average seawater temperatures in warm periods calculated over a range of temporal scales (from 1 day to the 90 warmest days) showed 1–2 °C difference between these two groups of localities (Fig. 2c).

### Recent thermal history at the study localities

The analysis of the recent thermal conditions (three months prior to the experiment) also revealed contrasting patterns between the different localities. The average *in-situ* mean daily temperature ranged between 16.85 °C ± 0.99 SD at Portofino to 21.8 °C ± 1.26 SD at Scandola (Fig. 2d–g). During this period, exposure to abnormally extreme temperatures occurred on 9 and 5 days at Medes and Scandola, respectively (Fig. 2d,e). By contrast, populations at Portofino and Kornati were never exposed to such extreme conditions during the three-month period prior to the experiment (Fig. 2f,g). Similarly, the maximum daily temperature reached in this period was highest at Medes (24.56 °C). A difference of almost 4 °C was evident between the warmest day at Medes and that at Portofino (20.55 °C), revealing the disparity of in *in-situ* thermal exposure experienced by the populations in the 3 month period prior to the experiment.

### Past thermal stress history (i.e., previous exposure to MHW-induced MMEs over the 1983–2017 period)

All eleven populations studied here were affected to some extent during previous MHW-induced MMEs reported to have occurred over the last three decades in the Mediterranean. Yet, the degree of impact that they exhibited varied greatly among them (Table S1). Specifically, Lighthouse (Portofino) was the only population that experienced a severe MME over the 1983–2017 period (in 1999), followed by the Palazzu and Palazzinu populations (Scandola), which experienced moderate mass mortalities in 2003. Conversely, low impacts were reported for Altare and Indiano (Portofino) in 1999, Gargallu (Scandola) in 2003, as well as all three populations from Medes in 2003 and both Kornati populations in 2009.

### Thermotolerance patterns in *P. clavata* populations in relation to their local thermal history

**Figure 3:**
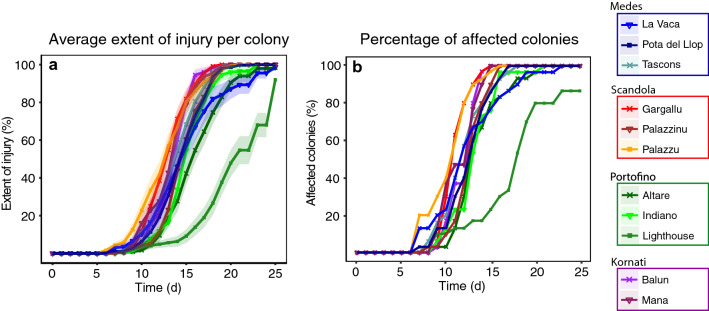
Extent of injury ± SE (**a**) and percentage of affected colonies (**b**) during the 25 days of exposure to thermal stress. Colors (i.e., blue, red, green and purple) correspond to the four localities while the different lines correspond to the 11 *P. clavata* populations analyzed.

The first signs of necrosis were evident for all populations in at least some colonies after one week exposed to 25 °C (Fig. 3a). At the end of the experiment (25 days), 94.5% of the colonies (312 out of 330) showed 100% tissue necrosis (dead colonies) while only 1.2% (4 out of 330) remained totally healthy (all from Lighthouse population) (Fig. 3b).

There were no differences in the survival probability across time of *P. clavata* colonies depending on their local thermal regime according to the results of the COX models (p > 0.05; Table S2). Specifically, the mean annual T of the sampled localities did not significantly affect the thermal response. Similarly, the results of the COX mixed models focusing on the effects of recent thermal history were similar, with no significant relationship between the thermal response of populations and the thermal conditions in the three months prior to the experiment (p > 0.05*;* Table S3). However, the results of the COX mixed models focusing on the effects of thermal stress history (defined as damage suffered during previous MHW-induced MMEs) showed a significant positive relationship between the thermotolerance of populations and degree of impact experienced in past MHW-induced MMEs (p < 0.01*;* Table S4)*.*

#### Thermotolerance across localities

**Figure 4:**
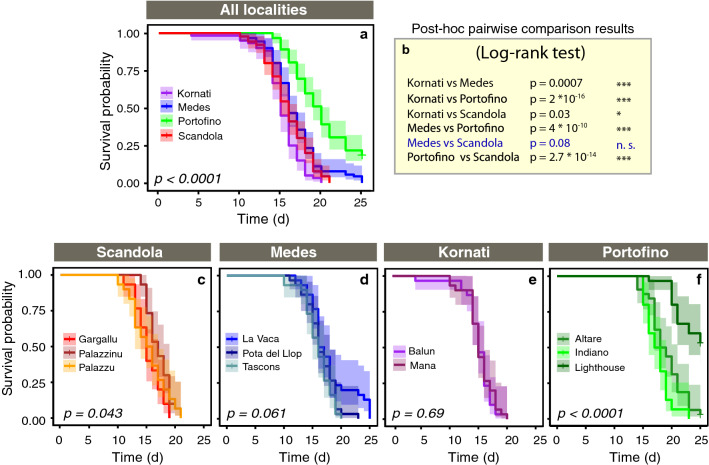
(**a**) Differences in survival probability across localities in *P. clavata* when exposed to thermal stress. (**b**) Post-hoc pairwise comparison across localities. (**c**–**f**). Differences in survival probability across populations (within localities).

Significant differences in the survival probability were found between the localities (p < 0.001) (Fig. 4a). The colonies from Portofino were significantly more resistant than any of the remaining three localities: Medes, Kornati or Scandola (Fig. 4b). Among the latter three less resistant localities, the survival probability of colonies from Scandola and Medes did not differ significantly but both were higher than that found at the most sensitive locality of Kornati (Fig. 4b).

#### Thermotolerance across populations (within localities)

Within localities, significant differences in the survival probability among populations were found only in two cases: between the populations at Palazzinu and Gargallu from Scandola (Fig. 4c), and between the three populations at Portofino (Fig. 4f). By contrast, the survival probability was the same among the populations at Medes (Fig. 4d) and Kornati (Fig. 4e). When looking at the overall pool of populations studied, the post-hoc pairwise comparison tests showed that 55% of the populations pairs (32 out of 58 possible combinations) did not differ significantly in their responses to thermal stress despite belonging in many cases to different localities (Table S5). For instance, the response of the Balun population (Kornati) was not only equivalent to that of Mana from the same locality, but also to that of other populations from different localities such as Palazzu and Gargallu (Scandola) or La vaca (Medes). Statistically significant differences in the responses to thermal stress were especially evident between the most resistant and sensitive populations (45% of the pairs). For example, for the most sensitive populations such as Balun (Kornati), Mana (Kornati) or Palazzu (Scandola), half of the colonies were affected by thermal stress only after 11–12 days of exposure to 25 °C, with 100% of the colonies dying in less than 21 days (Fig. 3). By contrast, the colonies from Lighthouse (Portofino), which was the most resistant population, took at least 18 days to show the same 50% effect, while exhibiting an average colony necrosis of only 55% ± 40 (mean ± SE) after 21 days.

### Intra-population variability in the responses to thermal stress

**Figure 5:**
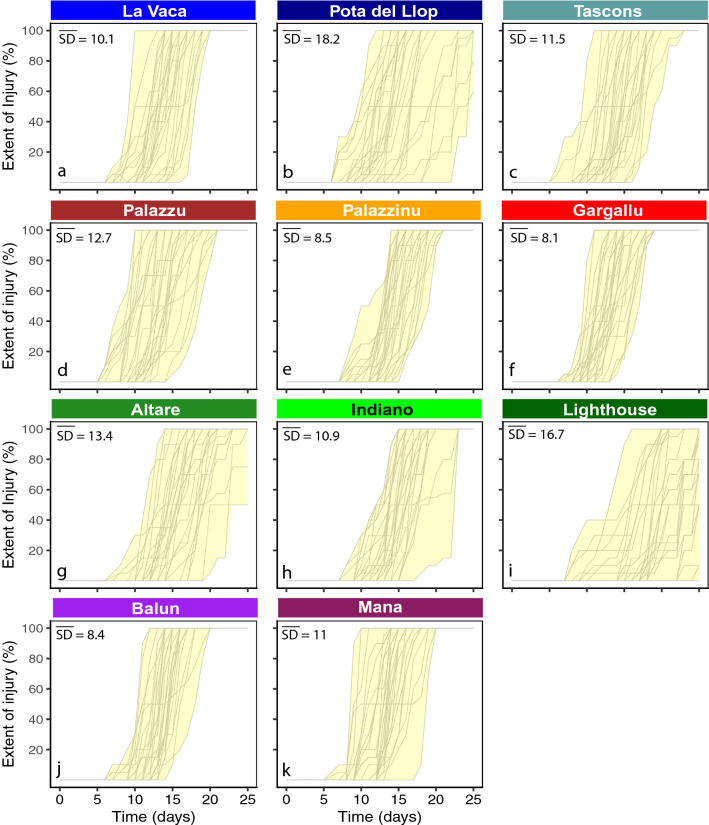
Intra-population variability observed in *P. clavata* populations exposed to heat stress. The response curve of each colony is shown in grey for each population ((**a**–**c**) Medes, (**d**–**f**) Scandola, (**g**–**i**) Portofino and (**j**–**k**) Kornati). A yellow ribbon has been added to visualize the overall variability in the population (encompassing the temporal span between the most resistant colony and the most sensitive colony). The mean daily SD of extent of injury among colonies calculated across the 25 days of the experiment is also shown for each plot.

Important differences in intra-population variability were observed among populations. Three populations (Palazzinu and Gargallu from Scandola and Balun from Kornati) showed low variability and a similar response in all the colonies (Fig. 5e,f,j). For these “low variability” populations, the mean daily SD of the % extent of injury among colonies across the 25 days of experiment was lower than 10%. By contrast, the individual response of colonies from other populations such as Pota del Llop (Medes) or Lighthouse (Portofino) was highly variable (Fig. 5b,i), with greater temporal differences in the onset of necrosis and mortality between sensitive and resistant colonies (with the latter occasionally unaffected). For these “highly variable” populations, the mean daily SD of the % extent of injury among colonies across the 25 days of experiment was higher than 15%. Finally, a third group including the majority of populations displayed intermediate intra-population variability. These “intermediate” populations (mean daily SD of 10–15% injury among colonies across the 25 days of experiment) were La Vaca (Medes), Tascons (Medes), Palazzu (Scandola), Altare (Portofino), Indiano (Portofino) and Mana (Kornati) (Fig. 5a,c,d,g,h,k).

## Discussion

In this study we carried out a comprehensive characterization of thermal stress sensitivity in *Paramuricea clavata* across several spatial and biological scales. In a common garden experiment including 11 populations from four localities with contrasting thermal regimes and separated by more than 1500 km of coast, a broader scale than previously addressed for this species, we demonstrate that local thermal regimes and recent thermal histories have limited influence in shaping the responses of populations to thermal stress. Together with the high sensitivity shown by most populations in the experiment, our results question the adaptive potential of *P. clavata* to the ocean warming that is predicted to occur over the next decades across most of its distribution range^58,59^. Nevertheless, the increased thermotolerance of extant colonies that had been previously exposed to MHW-induced MMEs, together with the significant inter-individual differences observed in the responses to thermal stress in some populations, suggests some potential capacity of *P. clavata* to tolerate or adapt to future conditions via adaptive phenotypic (intra or trans-generational acclimation processes) and/or genetic changes (adaptation via natural selection).

### Local thermal environment and thermal history *vs.* thermotolerance in *P. clavata*: exploring the role of local adaptation and acclimation

Like a previous study conducted at a smaller spatial scale in the NW Mediterranean (three localities separated by 500 km)^25^, our results did not reveal a relationship between thermal tolerance and the average local thermal environment of the source *P. clavata* populations. Moreover, the recent thermal history of the populations was not linked with increased resistance to thermal stress. Given the larger geographic and biological scope of the present study, our results further suggest that the response of *P. clavata* to thermal stress is not shaped by local adaptation to thermal regimes nor by rapid acclimation processes to warm conditions. Interestingly, these results contrast with the majority of the studies conducted to date for corals (e.g.,^10–13,16–19,60,61^) and other marine species (e.g.,^62–64^), which have typically found that living in warmer habitats or experiencing thermal pre-conditioning increases thermotolerance (but see^22–24,26^).

Conversely, our results suggest that processes other than local adaptation and acclimation to thermal regimes mainly drive the intra-specific variation in thermal stress response in *P. clavata*. These may include neutral evolutionary forces (i.e., genetic drift hampering local adaptation)^25,36^, food availability^30,31^ demographic factors (female *vs.* males or adult *vs.* juveniles)^36^, or the presence of temperature-dependent pathogens^32,33^. Nevertheless, a role for local thermal adaptation and/or thermal acclimation in shaping thermal stress responses in *P. clavata* should not be rejected entirely. Firstly, some studies suggest that local adaptation to high-frequency thermal variation (e.g., daily temperature range), which was not tested in our study, could be a more important driver of thermal tolerance than the average thermal regime (e.g.,^65^). Secondly, prior exposure to MHW-induced MMEs (considered here as a proxy for thermal stress history) could also modify the way in which populations respond to thermal stress by selecting genotypes with thermal resistance and/or the ability to rapidly acclimatize.

Our results showed a significant positive relationship between previous exposure to severe MHW-induced MMEs and greater thermotolerance. The Lighthouse population at Portofino was the most severely affected by a MHW-induced MME in 1999 (Table S1), and was the most resistant population in our experiment. Studies on scleractinian corals have shown that sites that experience extreme bleaching may experience reduced bleaching during subsequent events in comparison to unaffected sites (e.g.,^21,66^). However, in scleractinian corals, these patterns may be related to adaptation, acclimation, or re-assortments of their photosymbionts. However, *P. clavata* is an azooxanthellate species. Therefore, other hypotheses need be considered when attempting to explain the higher thermotolerance of the Lighthouse population. The first involves phenotypic plasticity and long-term acclimation; colonies impacted in 1999 may have been able to acclimate to the extreme warm conditions via phenotypic plasticity (e.g., via downregulating stress response genes such those associated with apoptotic signaling or through upregulation of heat shock proteins^18,67^), and have maintained this “more thermotolerant” phenotype over the years. In gorgonians, such long-term acclimation could also involve shifts of baseline expression of multiple colony genes that assist in coping with oxidative stress^8,35^. The long-term persistence of acclimation could contribute to population persistence by gaining time for genetic adaptation to occur^68^. Acclimated traits could have direct adaptive value if transmission via epigenetic mechanisms occurs^69–71^. However, while we cannot discard long-term acclimation having occurred at Lighthouse, the sensitive response to thermal stress in other *P. clavata* populations that had experienced exceptionally high temperatures in the three months prior to our experiment (e.g., Scandola), suggests a limited capacity for rapid acclimation in this species.

A second, non-mutually exclusive hypothesis to explain the higher thermotolerance of the Lighthouse population may lie in a higher proportion of thermotolerant genotypes. The 1999-MME could have caused a genetic bottleneck in which the less thermotolerant genotypes may have been lost. If so, the colonies remaining today in Lighthouse would be among the best suited to cope with experimental thermal stress applied. The existence of naturally occurring thermotolerant genotypes is consistent with the high inter-individual differences in thermotolerance observed in some populations in this study. Moreover, it would imply the existence of standing genetic variation among individuals in thermotolerance-related genes (e.g., single-nucleotide polymorphisms^72^), which would confer some adaptive potential to climate change for *P. clavata* via natural selection. However, the loss of sensitive genotypes and the potentially strong selection in favor of thermoresistant colonies that follows could lead to further reductions in population size, which may come at a cost such as an increase of demo-genetic stochasticity^73^. Indeed, reduced population size can lead to loss of genetic diversity via increased inbreeding and genetic drift^74–76^. Moreover, although strong adaptability to heat stress may be beneficial during MHWs, the surviving genotypes may be maladapted to the average local conditions^77^, which may also have physiological costs such as lower investment in reproductive effort^36^. Consequently, MHW-driven reductions in population size could increase extinction risk and hinder adaptive potential^78,79^. Finally, a third explanation of the higher resistance shown by the Lighthouse population could be related to environmental and physiological factors unrelated to the thermal stress history. For instance, a higher food availability during the summer months prior to the sampling and/or a lower investment in reproduction in comparison to the other populations could have led to a greater nutritional status of Lighthouse population during the experiment, lower energetic constraints and thus higher thermotolerance^29,30,80^. Unfortunately, the nutritional status of the populations was not estimated in our study. Therefore, whether energy reserves influenced the higher thermotolerance of Lighthouse population remains an open question. Nevertheless, the fact that other populations from the same area and depth (i.e., Altare and Indiano) did not exhibit a higher thermotolerance despite having likely been subjected to similar food conditions during summer, suggests that the nutritional status of populations was not the most decisive factor involved. In addition, previous experiments conducted with *P. clavata* populations sampled before and after the summer (therefore with different nutritional status^29^), showed that the nutritional status of colonies does not significantly affect thermotolerance when colonies are fed during the experiment^31^, as was the case in our experimental setup. Moreover, the energy reserves of colonies seem to play a very limited role in their thermotolerance when compared with other factors such as their antioxidant capacity^35^. Overall, these data support the higher thermotolerance observed in Lighthouse population as more likely being related to the thermal stress history than to other environmental or physiological factors influencing its energetic reserves.

Nevertheless, not all populations that were impacted by MHWs in the past exhibited high thermotolerance in our study. For instance, Palazzu and Palazzinu populations from Scandola, which experienced moderate MHW-driven mortalities in the past, were among the most sensitive populations in aquaria. This suggests that the effect of previous MHWs on *P. clavata* thermal response may be highly context-specific. Hence, further research should investigate whether previous exposure to MHW conditions could improve population responses to climate change in this species.

### Thermotolerance in *P. clavata*: searching for resistant colonies and populations

In this study we have confirmed that the 25 °C lethal threshold proposed for *P. clavata* by^25^ is a lethal temperature for this species across most part of its distributional range. Concerningly, this temperature is becoming common during warm summers in a large part of the Mediterranean^81^. Hence, with more frequent and severe MHWs projected for the next decades, the exposure of *P. clavata* populations to detrimental environmental conditions will become more common, especially in the shallower part (0–50 m depth) of its bathymetric distribution range^58,59^. Consequently, many *P. clavata* populations could face collapse trajectories, with likely detrimental consequences for the functioning of the associated coralligenous assemblages^40^. Whether deep *P. clavata* populations will provide a viable reproductive source for its shallow counterparts following disturbance (the deep refugia hypothesis;^82^) remains unknown. Some evidence indicates that deep *P. clavata* populations could safeguard genetic diversity in the context of climate change^83^. However, a growing body of evidence suggests that the deep refugia hypothesis may not hold for *P. clavata* and other gorgonian species in most situations because: (1) thermal anomalies may impact deep populations as well (e.g., down to 50–100 m in some cases^27,84^), (2) there needs to be high connectivity between shallow and deep populations, which may be difficult due to physical (e.g., currents) and biological (e.g., low dispersal capacity) constraints^37,45,46,85^; and (3) “foreign” recruits could be better adapted to the deep conditions so that their fitness is reduced in shallow environments, as observed in the Mediterranean precious coral *C. rubrum*^24^. Consequently, it is likely that deep populations of *P. clavata* will not act as a source of recolonization for shallow habitats. Active restoration to aid the regeneration of impacted populations and thereby accelerate ecosystem recovery could be desirable. In the context of climate change, successful restoration actions should aim to identify colonies that are tolerant to current and future environmental conditions, particularly with regard to seawater temperature^86^.

Within this framework, we have screened the thermal tolerance of 11 populations from four regions and different thermal regimes, and found that only a small proportion of colonies (5.5%) and populations (9%) were able to survive a 25 days exposure to 25 °C. These results showcase the high sensitivity of *P. clavata* to this temperature across its distributional range, which challenges the feasibility of successful restoration actions in the face of climate change. It seems that contrarily to what may have been thought, selecting colonies from populations dwelling in warmer environments to restore potentially damaged habitats will not likely provide any benefit in comparison with selecting populations from other cooler thermal environments. Nevertheless, high levels of intra-population variability were found amongst colonies throughout the experiment in some populations (i.e., Pota del Llop or Lighthouse). In these highly variable populations, at least some colonies were able to withstand acute heat stress (i.e., 25 °C) during relatively long periods of time (some even during the 25 days of experiment). This is a strong-enough resistance to withstand the heat stress conditions observed during previous MHW-induced MMEs in the Mediterranean^87^, and at least some of the MHW conditions expected at their living depths during the next decades^58^. Consequently, such high inter-colony variability, could be key for an effective adaptation of *P. clavata* populations to climate change and should receive further attention from both conservation and restoration perspectives.

Future and ongoing research combining the thermotolerance data gathered in this study with analyses of differential gene expression will shed light on whether the inter- and intra-population variability in thermotolerance observed here is the result of acclimation due to transcriptional frontloading or differential capacity for gene expression plasticity^88^. In addition, the ongoing analyses of whole genome re-sequencing data using the recently assembled reference genome of *P. clavata*^89^, and the comparison of “resistant” vs. “vulnerable” individuals identified in this experiment, will allow the exploration of eco-evolutionary processes and potential genetic factors involved in the differential responses of colonies and populations to thermal stress. Finally, the combination of the thermotolerance data gathered in this study with ongoing analyses of *P. clavata* microbiome will allow to determine if microorganisms may have played an active role in the observed thermal stress susceptibility of *P. clavata* [Bonacolta et al. *in prep*]. Gathering this information at the transcriptomic, genomic and microbiome scales is essential to complement the results of the present study and to guide conservation and restoration efforts for *P. clavata* in the Mediterranean Sea.

## Supplementary Information

Supplementary Information.

## Data Availability

The datasets used and/or analyzed during the current study available from the corresponding author on reasonable request.
