# Supplementary material for: Exploring the response of a key Mediterranean gorgonian to heat stress across biological and spatial scales
Source: Sci Rep. 2022 Dec 6;12:21064. doi: 10.1038/s41598-022-25565-9 (PMC9726941; doi:10.1038/s41598-022-25565-9)

Electronic supplementary material

Exploring the response of a key Mediterranean gorgonian to heat stress across biological and spatial scales

Gómez-Gras D*[1,2,3], Bensoussan, N[1,4], Ledoux JB[5], López-Sendino P[1], Cerrano C[6,7,8,9], Ferretti E[9], Kipson S[10,11], Bakran-Petricioli T[10], Serrao EA[12] , Paulo D[12], Coelho MAG[12], Pearson GA[12], Boavida J[4], Montero-Serra I[2], Pagès-Escolà M[2], Medrano A[2], López-Sanz A[1] Milanese M[13], Linares C[2.3], Garrabou J[1,3].

1. Departament de Biologia Marina, Institut de Ciències del Mar (CSIC), Barcelona, Spain

2. Departament de Biologia Evolutiva, Ecologia i Ciències Ambientals, Universitat de Barcelona (UB), Barcelona, Spain.

3. Institut de Recerca de la Biodiversitat (IRBio), Universitat de Barcelona (UB), Barcelona, Spain.

4. University of Aix-Marseille, University of Toulon, CNRS, IRD, Mediterranean Institute of Oceanography (MIO) UTM 110, Marseilles, France

5. CIIMAR/CIMAR, Centro Interdisciplinar de Investigação Marinha e Ambiental, Universidade do Porto, Porto, Portugal.

6. Dipartimento di Scienze della Vita e dell'Ambiente (DiSVA), Università Politecnica delle Marche, , Ancona, Italy

7. Consorzio Nazionale Interuniversitario per le Scienze del Mare (CoNISMa), Rome, Italy

8. Stazione Zoologica Anton Dohrn, Naples, Italy

9. Reef Check Italia Onlus, Ancona, Italy

10. Department of Biology, Faculty of Science. University of Zagreb, Zagreb, Croatia

[11] SEAFAN – marine research & consultancy, Zagreb, Croatia

12. CCMAR, University of Algarve, Faro, Portugal

13. Studio Associato GAIA s.n.c., Genoa, Italy

Table S1. Previous exposure of the selected populations to MHW-induced MMEs

Table S2. Results from the Cox mixed-effects model examining survival in *P. clavata* populations according to their local average thermal regimes.

Table S3. Results from a Cox mixed-effects model examining survival in *P. clavata* populations according to their recent thermal history (i.e., thermal conditions in the three months prior to the experiment).

Table S4. Results from a Cox mixed-effects model examining survival in *P. clavata* populations according to their thermal stress history (i.e., previous experienced MHW-induced MMEs).

Table S5. Post-Hoc pair-wise comparison (log-rank test) of the survival results

Figure S1. Temperature time-series of the studied localities

Figure S2. Sampling procedure

Figure S3. Polyp activity during acclimation week

Figure S4. Experimental design (a) and setup (b).

Supplementary Appendix 1. Information on the sampled Sagres population*; Country, location, ID, coordinates and sampling depth.

Supplementary Appendix 2. Thermotolerance results of the *Sagres population.

Table S1. Previous exposure of the selected populations to warming-induced MMEs. Information extracted from the T-MEDnet mortality database from 1987 to 2017 (Garrabou et al. 2019).

Year	Mediterranean region	Sub-basin	Locality	Population	Long.	Lat.	Depth (m)	% of affected colonies
1999	North-West Mediterranean	Liguro-Provençal	Portofino	Lighthouse	9° 13' 8.45" E	44° 17' 55.14" N	35-37	Severe (> 60%)
1999	North-West Mediterranean	Liguro-Provençal	Portofino	Altare	9° 10' 4.12" E	44° 18' 32.10" N	35-37	Low (< 30%)
1999	North-West Mediterranean	Liguro-Provençal	Portofino	Indiano	9° 10' 0.80" E	44° 18' 44.9" N	35-37	Low (< 30%)
2003	North-West Mediterranean	Liguro-Provençal	Corsica	Palazzu	8° 33' 4.93" E	42° 22' 42.64" N	23-26	Moderate (> 30 / < 60%)
2003	North-West Mediterranean	Liguro-Provençal	Corsica	Palazzinu	8° 33' 8.64" E	42° 22' 34.88" N	23-26	Moderate (> 30 / < 60%)
2003	North-West Mediterranean	Liguro-Provençal	Corsica	Gargallu	8° 32' 3.82" E	42° 22' 18.62" N	24-27	Low (< 30%)
2003	North-West Mediterranean	Liguro-Provençal	Medes	Pota del Llop	3º 13' 34.7" E	42º 2' 52.97" N	18 - 20	Low (< 30%)
2003	North-West Mediterranean	Liguro-Provençal	Medes	Tascons	3º 13 '31.4' 'E	42º 2' 58.92" N	15 -17	Low (< 30%)
2003	North-West Mediterranean	Liguro-Provençal	Medes	La Vaca	3° 13' 36.8" E	42° 2' 31.88" N	15 – 17	Low (< 30%)
2009	Central Mediterranean	Adriatic Sea	Kornati	Balun	15° 15'18" E	43° 48' 14" N	33 - 36	Low (< 30%)
2009	Central Mediterranean	Adriatic Sea	Kornati	Mana	15° 15' 59" E	43° 48'01" N	35	Low (< 30%)

References

Garrabou J, Gómez-Gras D, Ledoux J-B, Linares C, Bensoussan N, López-Sendino P, et al. (2019) Collaborative Database to Track Mass Mortality Events in the Mediterranean Sea. Front. Mar. Sci. 6:707. https://doi.org/10.3389/fmars.2019.00707

Table S2. Results from a Cox mixed-effects model examining survival in *P. clavata* populations according to their average local thermal regimes.

Models	Fixed effects	Estimate	Std. Error	z value	*P-value*	AIC
**Mortality ~ Annual Mean T+ (1|Locality/Population)**	Annual Mean T	0.77	1.06	073	0.47	122.38
	**Random effects**	**St. deviation**	**Variance**			
	Locality/ Population	0.63	0.4			
	Locality	0.54	0.3			

Table S3. Results from a Cox mixed-effects model examining survival in *P. clavata* populations according to their recent thermal history (i.e., thermal conditions in the three months prior to the experiment. * The number of extreme heat days was calculated as the number of days with temperatures over the inter-annual percentile 90th based on the local climatology, and considering only days with a daily temperature average of at least 23 ºC.

Models	Fixed effects	Estimate	Std. Error	z value	*P-value*	AIC
**Mortality ~ N of extreme heat days + (1|Locality/Population)**	N of extreme heat days	0.04	0.1	0.39	0.69	122.1
	**Random effects**	**St. deviation**	**Variance**			
	Locality/ Population	0.63	0.39			
	Locality	0.6	0.36			

Table S4. Results from a Cox mixed-effects model examining survival in *P. clavata* populations according to their thermal stress history (i.e., degree of damage during previously experienced MHW-induced MMEs).

Models	Fixed effects	Estimate	Std. Error	z value	*P-value*	AIC
**Mortality ~ Degree of damage during previous MHW-induced MMEs + (1|Locality/Population)**	Severity (MME)	-0.84	0.19	-4.53	$5.8 * 10^{-6}$	
	**Random effects**	**St. deviation**	**Variance**			
	Locality/ Population	0.25	0.06			
	Locality	0.61	0.37			

Table S5. Post-Hoc pair-wise comparison (log-rank test) of the survival results among populations. Significant results (*$p \leq 0.05$*) have been highlighted in red, while non-significant results (*$p > 0.05$*) appear in blue.

	Altare	Balun	Gargallu	Indiano	Lighthouse	Mana	Palazzinu	Palazzu	Pota.	Sagres	Tascons
**Balun**	0.009	-	-	-	-	-	-	-	-	-	-
**Gargallu**	$5 * 10^{-5}$	0.095	-	-	-	-	-	-	-	-	-
**Indiano**	0.71	0.016	0.0003	-	-	-	-	-	-	-	-
**Lighthouse**	$4.8 * 10^{-5}$	$4.4 * 10^{-9}$	$1.1 * 10^{-9}$	$1.1 * 10^{-9}$	-	-	-	-	-	-	-
**Mana**	0.009	0.78	0.37	0.025	$1.2 * 10^{-8}$	-	-	-	-	-	-
**Palazzinu**	0.17	0.09	0.009	0.3	$2 * 10^{-8}$	0.16	-	-	-	-	-
**Palazzu**	0.001	0.14	0.9	0.0008	$7.7 * 10^{-10}$	0.23	0.004	-	-	-	-
**Pota**	0.56	0.26	0.48	0.9	$4.8 * 10^{-5}$	0.21	0.68	0.031	-	-	-
**Tascons**	0.18	0.11	0.003	0.49	$4.1*10^{-8}$	0.17	0.79	0.005	0.68	$2.1*10^{-10}$	-
**Vaca**	0.063	0.54	0.063	0.21	$4.3*10^{-8}$	0.52	0.68	0.06	0.42	$1.1*10^{-10}$	0.66

Figure S1. Temperature time series used to explore the in-situ thermal regimes of the studied localities.

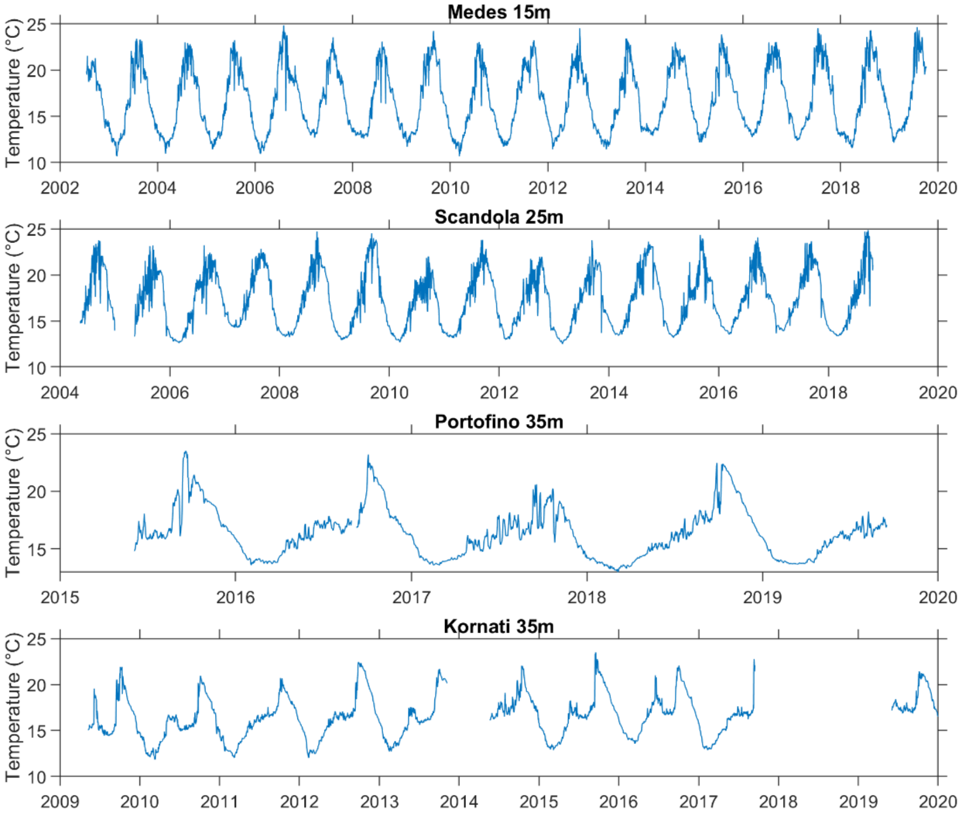

Figure S2. Sampling procedure

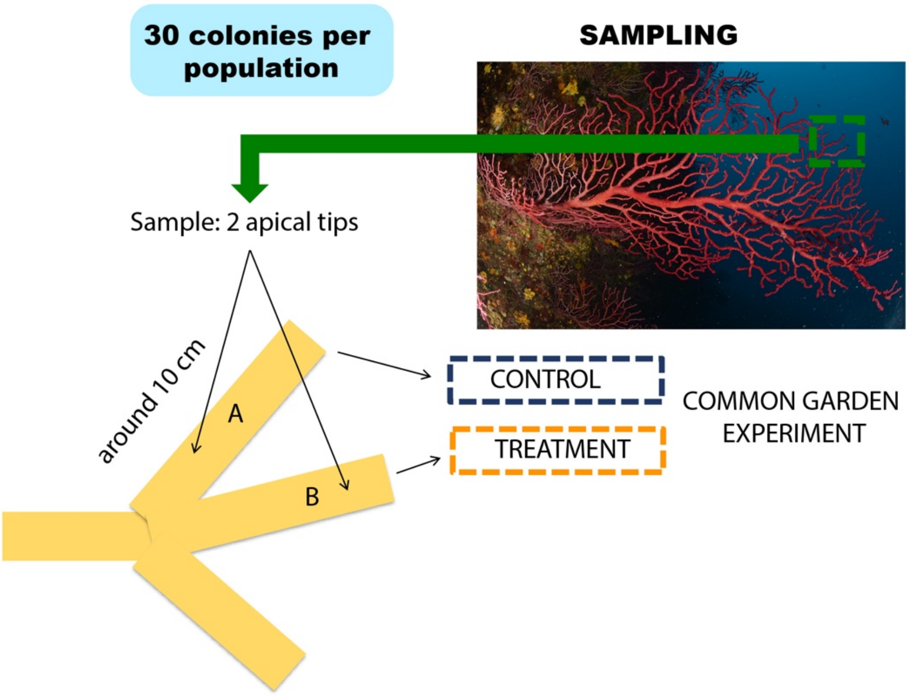

Figure S3. Polyp activity during acclimation week.

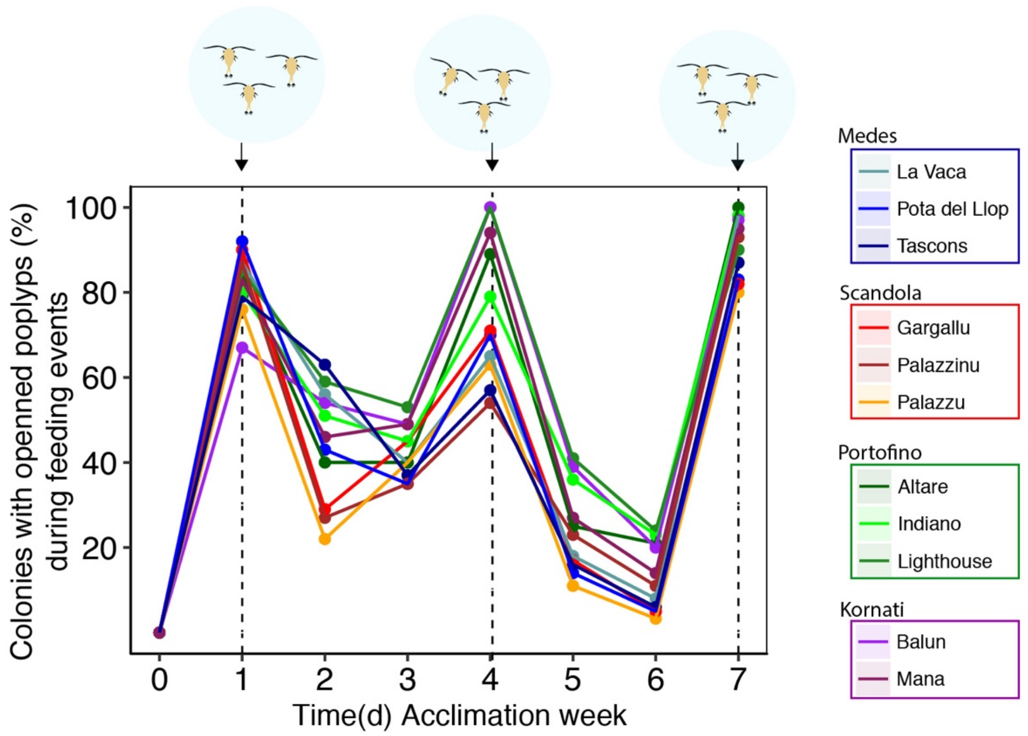

Figure S4. Experimental design (a) and setup (b)

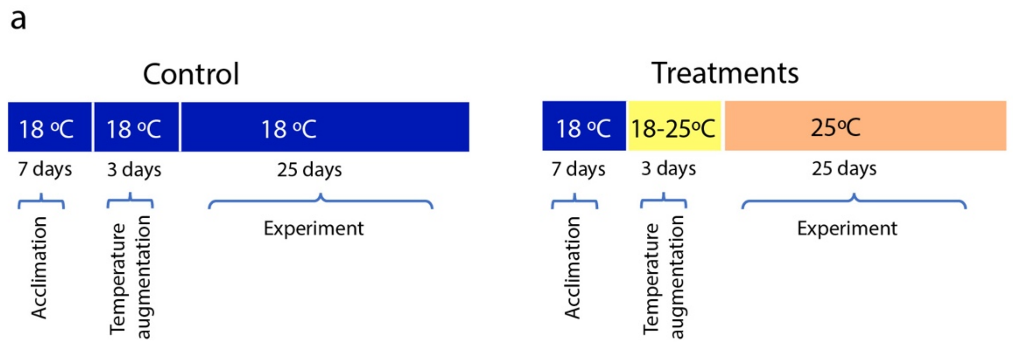

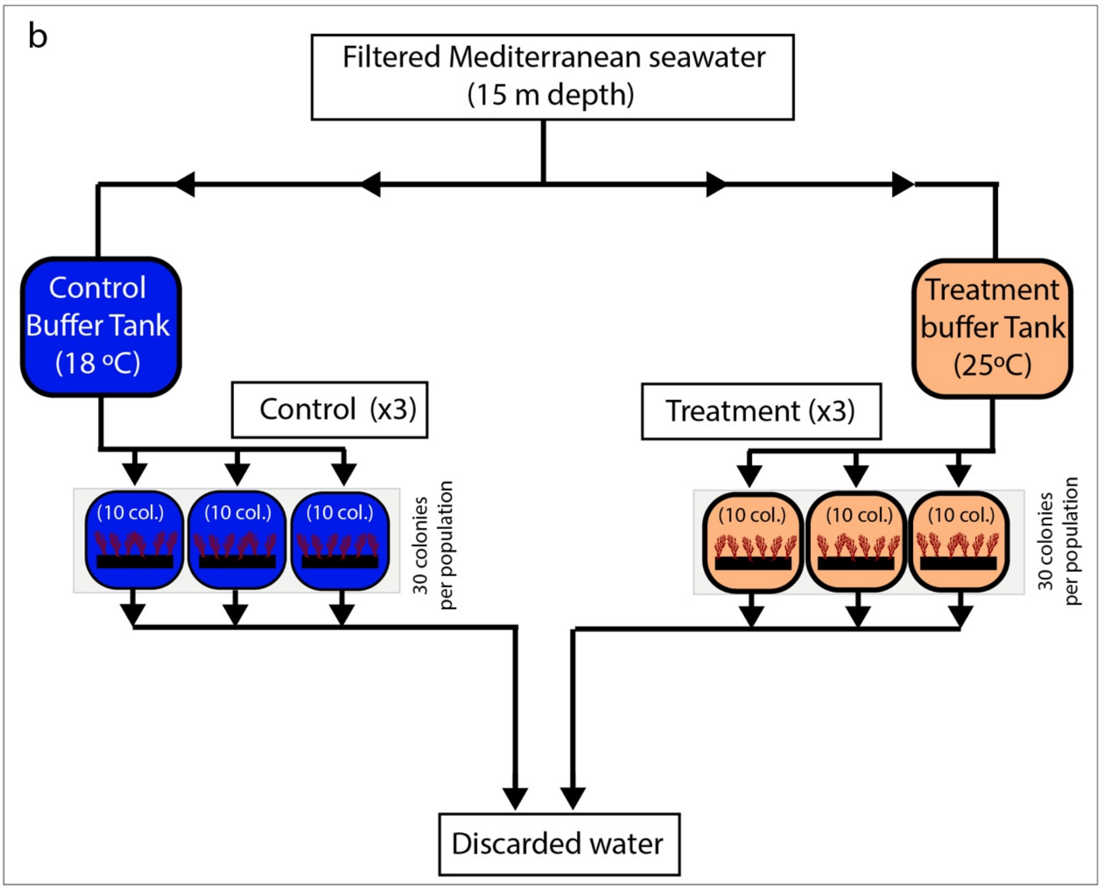

Supplementary appendix 1. Information on the sampled Sagres population*; Country, location, ID, coordinates and sampling depth.

Country	Location (Region)	Population ID	Longitude	Latitude	Depth (m)
Portugal	Sagres (Algarve)	Sagres	8° 55' 28.35" W	37° 0' 43.66" N	15

Supplementary appendix 2. Thermotolerance results of the Sagres population*, in comparison to the eleven populations of *P. clavata* tested in this study.

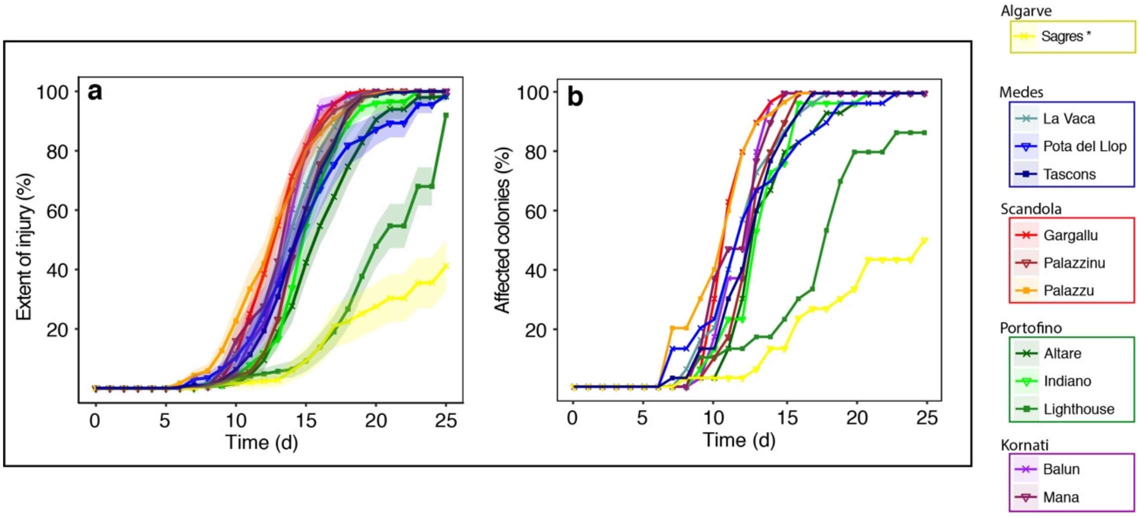

Supplement: Supplementary file 1 — Supplementary Information. [file 41598_2022_25565_MOESM1_ESM.pdf]
